# Fenvalerate Induces Airway Epithelial Injury Through CHRM3-Associated NF-κB Activation

**DOI:** 10.3390/ijms27209201

**Published:** 2026-10-16

**Authors:** Yuxin Yang, Jixin Li, Tong Liu, Mingyang Sheng, Jiatong Liu, Zidong Yang, Hongbo Chu, Ailing Jia, Wei Xu

**Affiliations:** College of Pharmacy, Changchun University of Chinese Medicine, Changchun 130117, China

**Keywords:** chronic obstructive pulmonary disease, fenvalerate, machine learning, single-cell RNA sequencing, CHRM3, NF-κB signaling pathway, inflammation

## Abstract

Fenvalerate (FEN) is a widely used pyrethroid insecticide with potential respiratory toxicity; however, its contribution to chronic obstructive pulmonary disease (COPD)-related pathological processes remains unclear. We investigated molecular mechanisms linking FEN exposure to airway epithelial injury. COPD transcriptomic datasets were integrated with predicted FEN targets, and candidate genes were prioritized using machine-learning algorithms. Immune infiltration and single-cell transcriptomic analyses were used to examine immune associations and cell-type-specific expression. Molecular docking and molecular dynamics simulations assessed potential FEN-target interactions, followed by validation in BEAS-2B human bronchial epithelial cells. Intersecting FEN-COPD genes were enriched in inflammatory, immune-regulatory, neuroactive ligand-receptor, and epithelial injury-related pathways. Eight candidate genes showed distinct expression patterns across airway epithelial and immune cell populations. Computational analyses supported a potentially stable FEN-CHRM3 interaction. In BEAS-2B cells, FEN reduced viability, increased IL-6 and IL-8 secretion, promoted apoptosis, upregulated CHRM3, and enhanced IκBα and p65 phosphorylation. Pharmacological inhibition of CHRM3 or NF-κB partially attenuated these responses. Thus, FEN-induced epithelial inflammation and apoptosis may involve CHRM3-associated NF-κB activation, providing mechanistic insight into pesticide-related respiratory toxicity.

## 1. Introduction

Chronic obstructive pulmonary disease (COPD) is one of the leading causes of morbidity and mortality worldwide and imposes a substantial burden on healthcare systems. It is primarily characterized by persistent respiratory symptoms, progressive airflow limitation, chronic airway inflammation, small airway remodeling, and destruction of lung parenchyma [1,2]. Although cigarette smoking remains the best-established risk factor for COPD, accumulating evidence suggests that COPD is a highly heterogeneous chronic airway disease whose onset and progression are influenced by multiple factors, including environmental and occupational exposures, early-life events, socioeconomic conditions, and host susceptibility [1,3]. This recognition has important clinical and public health implications, as a substantial proportion of patients with COPD have no history of heavy smoking, and non-smoking-related environmental risk factors remain underrecognized in current prevention strategies. Therefore, identifying modifiable environmental exposures and elucidating their roles in airway epithelial injury, immune dysregulation, and airway remodeling is essential for improving COPD risk stratification and enabling early prevention.

Pyrethroid insecticides (PYRs) are a class of synthetic insecticides derived from natural pyrethrins and have been widely used since the 1970s. Compared with many traditional organophosphate and organochlorine pesticides, PYRs exhibit high insecticidal potency, broad-spectrum activity, and relatively low acute toxicity to mammals and birds; consequently, they are extensively applied in agriculture, forestry, animal husbandry, aquaculture, household pest control, and public health [4,5]. PYRs account for a substantial proportion of the global pesticide market [6]. However, with their increasing use, human exposure via contaminated soil, water, food, indoor dust, and inhalable particulate matter has increased. Although PYRs are generally considered to have lower acute toxicity than conventional pesticides, accumulating evidence indicates that long-term, repeated, or low-dose exposure may still exert adverse effects on multiple organ systems, including the nervous, immune, reproductive, and respiratory systems [7,8].

The respiratory system may be particularly susceptible to PYRs, as the airway epithelium serves as the primary interface between inhaled environmental pollutants and the host immune system. In COPD, repeated epithelial injury and impaired repair capacity amplify oxidative stress, inflammatory cell recruitment, excessive mucus production, and extracellular matrix remodeling, thereby driving persistent airflow limitation and disease progression [2]. Experimental studies have demonstrated that exposure to PYRs can induce oxidative stress, inflammatory activation, disruption of epithelial barrier integrity, mitochondrial dysfunction, and immune dysregulation [9]. These biological processes substantially overlap with the established pathogenic mechanisms of COPD. However, compared with well-recognized air pollutants such as particulate matter, biomass smoke, and occupational dust, pesticides have received relatively limited attention as potential contributors to chronic airway diseases. The molecular mechanisms linking exposure to PYR insecticides with COPD remain poorly understood, and it remains unclear whether PYR-associated molecular targets intersect with key pathogenic pathways involved in COPD development.

Fenvalerate (FEN) is a representative type II PYR insecticide used in agricultural and household pest control. Environmental monitoring studies have detected FEN residues in soil, water, and agricultural products, indicating multiple potential routes of human exposure [10,11,12]. Experimental studies have linked FEN exposure to oxidative stress, inflammatory responses, endoplasmic reticulum stress, mitochondrial dysfunction, and apoptosis [13,14,15]. In an inhalation study in rats, a formulated FEN preparation increased biochemical markers of lung injury and produced inflammatory cell infiltration, epithelial hypertrophy, alveolar edema, and vascular congestion [16]. These observations suggest biological plausibility for FEN-associated airway injury, but they do not establish that FEN causes COPD. The molecular overlap between FEN toxicity and COPD-related airway pathology therefore warrants investigation.

At the mechanistic level, the onset and progression of COPD involve complex interactions among multiple biological processes, including environmental exposures, airway epithelial dysfunction, innate and adaptive immune responses, oxidative stress, protease-antiprotease imbalance, cellular senescence, and tissue remodeling. Many of these pathways overlap with those implicated in pesticide-induced toxic responses. This convergence of biological mechanisms provides a theoretical rationale for investigating whether FEN-associated molecular targets intersect with COPD-related transcriptional alterations and changes in the immune microenvironment. However, current evidence remains fragmented. Existing toxicological studies have primarily focused on describing pulmonary injury and other organ-specific toxic effects following FEN exposure [16,17], whereas the molecular mechanisms linking FEN exposure to COPD-associated airway epithelial injury remain largely unexplored. In particular, FEN-associated molecular targets have not been systematically integrated with COPD transcriptomic alterations, immune-microenvironment characteristics, and experimental validation within a unified disease-specific mechanistic framework. This knowledge gap provided the rationale for the present integrative bioinformatics and experimental investigation.

Therefore, this study investigated molecular links between predicted FEN targets and COPD-related transcriptional alterations using an integrated systems-biology framework. COPD transcriptomic datasets from the Gene Expression Omnibus (GEO) were integrated with predicted FEN targets, and multiple machine-learning algorithms were used to prioritize candidate genes. Immune associations and cell-type-specific expression patterns were then examined using immune infiltration and single-cell RNA sequencing (scRNA-seq). Molecular docking and molecular dynamics (MD) simulations were used to evaluate potential FEN–target interactions. Finally, BEAS-2B human bronchial epithelial cells were used to assess cell viability, inflammatory cytokine secretion, NF-κB signaling, and apoptosis after repeated FEN exposure and pharmacological inhibition. This combined approach was designed to generate and experimentally examine a mechanistic hypothesis for FEN-associated airway epithelial injury rather than to infer clinical causation of COPD.

## 2. Results

### 2.1. Identification of Intersecting Targets and Enrichment Analysis

**Figure 1:**
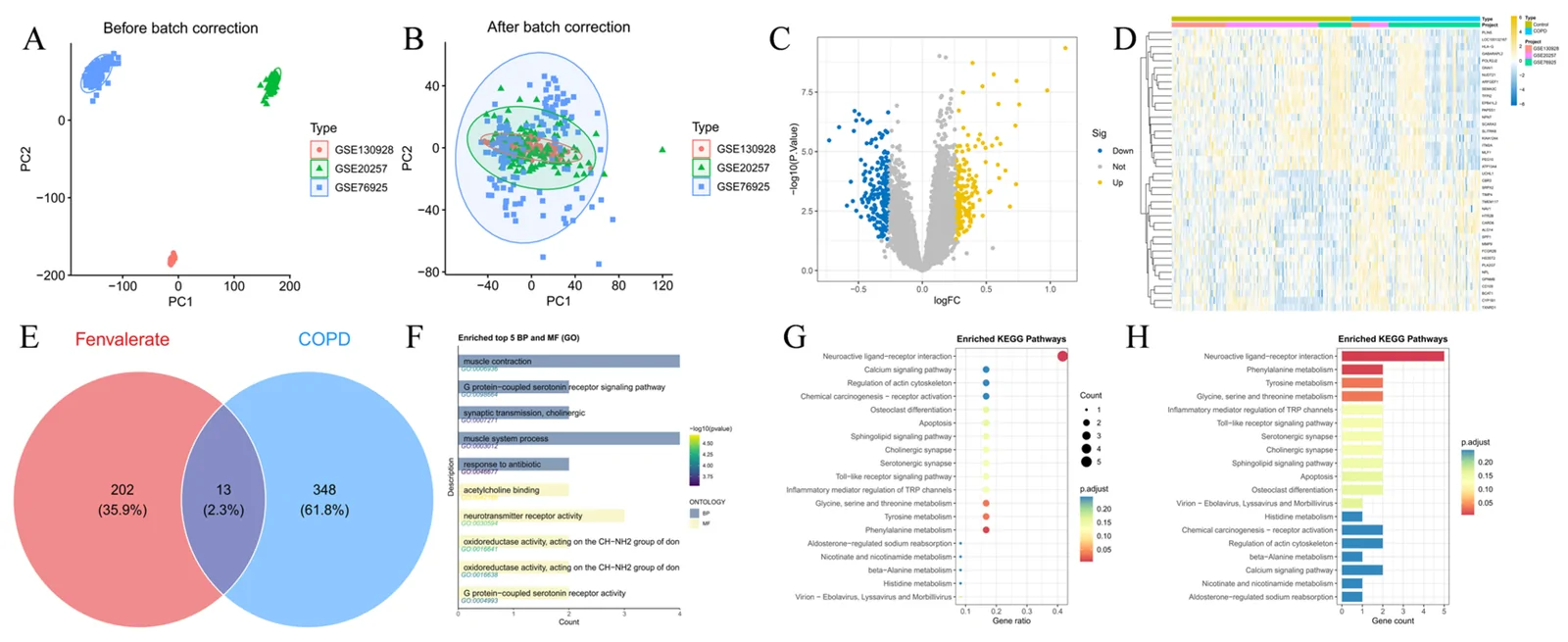
Identification and enrichment analysis of intersecting targets between FEN and COPD. (**A**) PCA plot before batch correction. (**B**) PCA plot after batch correction. (**C**) Volcano plot of DEGs. (**D**) Heatmap of DEGs. (**E**) Venn diagram showing overlapping targets between FEN and COPD. (**F**) GO enrichment analysis: blue bars indicate BP and yellow bars indicate MF. The *x*-axis represents gene count, and color intensity reflects *p*-values. (**G**) KEGG enrichment bubble plot: the *x*-axis indicates Gene Ratio and the *y*-axis represents pathway names. Bubble size corresponds to gene count, and color indicates adjusted *p* values. (**H**) KEGG enrichment bar plot: the *x*-axis shows gene count and the *y*-axis represents pathway names, with colors indicating adjusted *p* values.

A total of 104, 21, and 110 FEN-related targets were obtained from the SwissTargetPrediction, Similarity Ensemble Approach, and SuperPred databases, respectively. After integration and deduplication, 215 potential targets were identified (Supplementary Table S1). Three independent COPD-related transcriptomic datasets representing alveolar macrophages (GSE130928), small airway epithelium (GSE20257), and lung tissue (GSE76925) were included to capture complementary pulmonary compartments involved in COPD pathobiology. After normalization and batch-effect correction, the datasets were integrated for downstream analysis. PCA showed clear separation of samples before correction (Figure 1A), whereas samples overlapped after ComBat adjustment (Figure 1B), indicating effective removal of batch effects. Differential expression analysis using limma identified 361 COPD-associated differentially expressed genes. The volcano plot illustrated the distribution of upregulated and downregulated genes (Figure 1C), and the heatmap demonstrated clear separation between COPD and control samples (Figure 1D). Intersection analysis between COPD-associated differentially expressed genes and FEN targets identified 13 common genes (Figure 1E), which were considered potential key targets of FEN in COPD.

GO enrichment analysis showed that these genes were mainly enriched in biological process and molecular function categories, with no significant enrichment in cellular component terms (Figure 1F). At the biological process level, enriched terms included muscle contraction, G protein-coupled serotonin receptor signaling, cholinergic synaptic transmission, and response to antibiotics. At the molecular function level, genes were primarily enriched in acetylcholine binding, neurotransmitter receptor activity, and G protein-coupled serotonin receptor activity. KEGG pathway analysis revealed significant enrichment in neuroactive ligand-receptor interaction, calcium signaling, regulation of actin cytoskeleton, and osteoclast differentiation pathways (Figure 1G,H). Among these, neuroactive ligand-receptor interaction showed the highest enrichment. In addition, calcium signaling and cytoskeleton-related pathways, as well as Toll-like receptor and TRP channel-associated pathways, were also enriched, suggesting potential involvement in airway function and inflammatory regulation.

### 2.2. Identification, Differential Expression, Chromosomal Localization, and Expression Correlation Analysis of Core Genes

**Figure 2:**
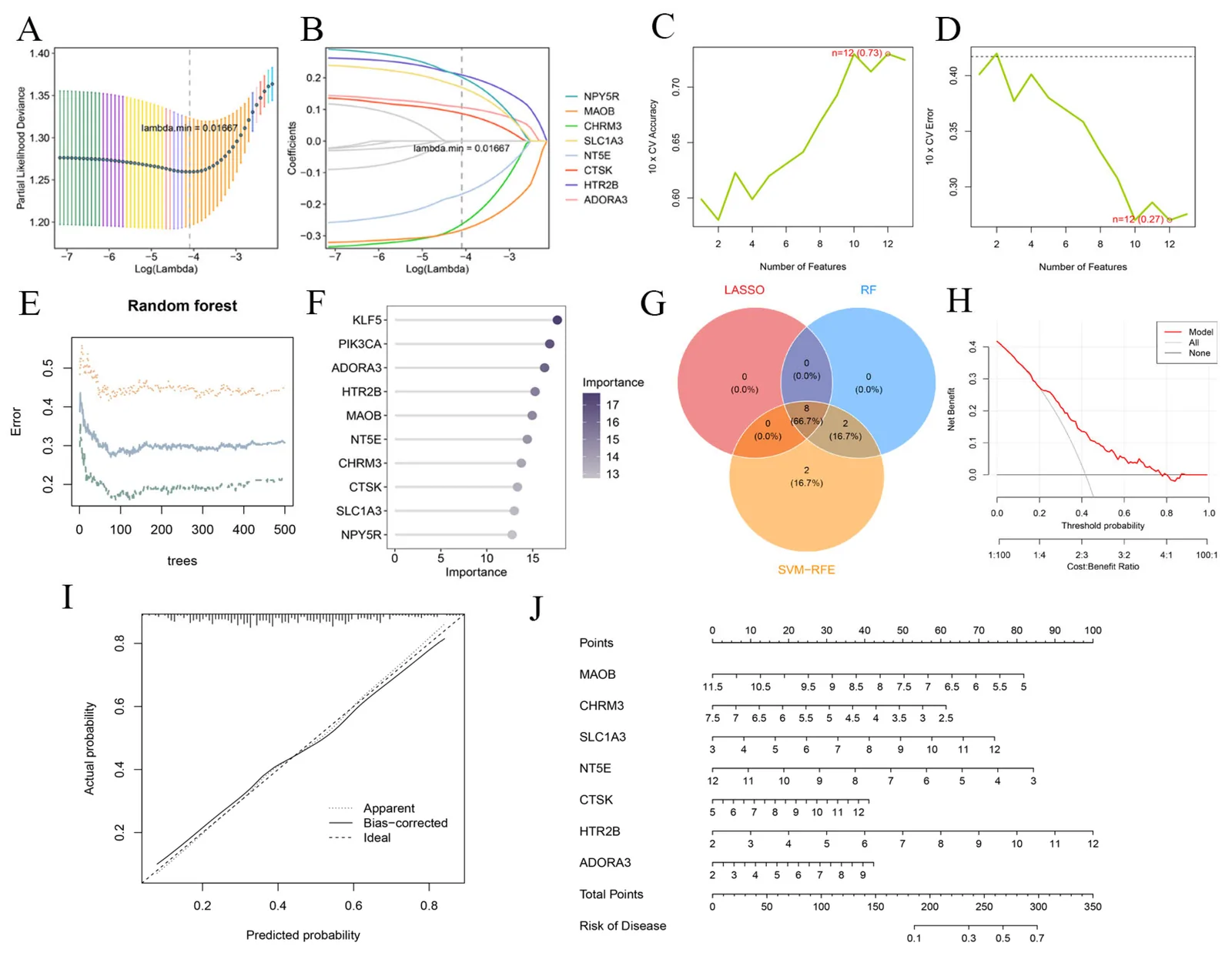
Identification and validation of core genes involved in FEN-induced COPD. (**A**,**B**) LASSO regression: (**A**) Ten-fold cross-validation for selection of the optimal λ; (**B**) Coefficient profiles at different λ values. The vertical dashed lines in panels A and B indicate the selected λ value (λmin = 0.01667). (**C**,**D**) SVM analysis: (**C**) Model accuracy versus number of features; (**D**) Error rate versus number of features, with the optimal feature set indicated. (**E**,**F**) RF model: (**E**) Classification error rate across different numbers of trees; (**F**) Feature importance ranking. (**G**) Venn diagram showing the intersection of genes identified by LASSO, SVM, and RF. (**H**) Decision curve analysis (DCA) of the final model. (**I**) Calibration curve of the model. The dotted, solid, and dashed lines represent apparent calibration, bias-corrected calibration, and ideal calibration, respectively. (**J**) Nomogram based on the selected genes for estimating COPD probability.

LASSO regression (Figure 2A,B), SVM (Figure 2C,D), and RF (Figure 2E,F) were used to prioritize candidate genes. Eight genes were selected by all three algorithms: NPY5R, MAOB, CHRM3, SLC1A3, NT5E, CTSK, HTR2B, and ADORA3 (Figure 2G). Decision-curve and calibration analyses are shown in Figure 2H,I, respectively, and a nomogram based on the selected genes for estimating COPD probability is presented in Figure 2J. Differential-expression and chromosomal-localization results are provided in Supplementary Figure S1. Because these analyses were conducted in the integrated discovery cohort, the genes were treated as candidates for mechanistic investigation rather than externally validated diagnostic biomarkers.

### 2.3. Multi-Model Comparison and SHAP Analysis of Candidate-Gene Contributions

**Figure 3:**
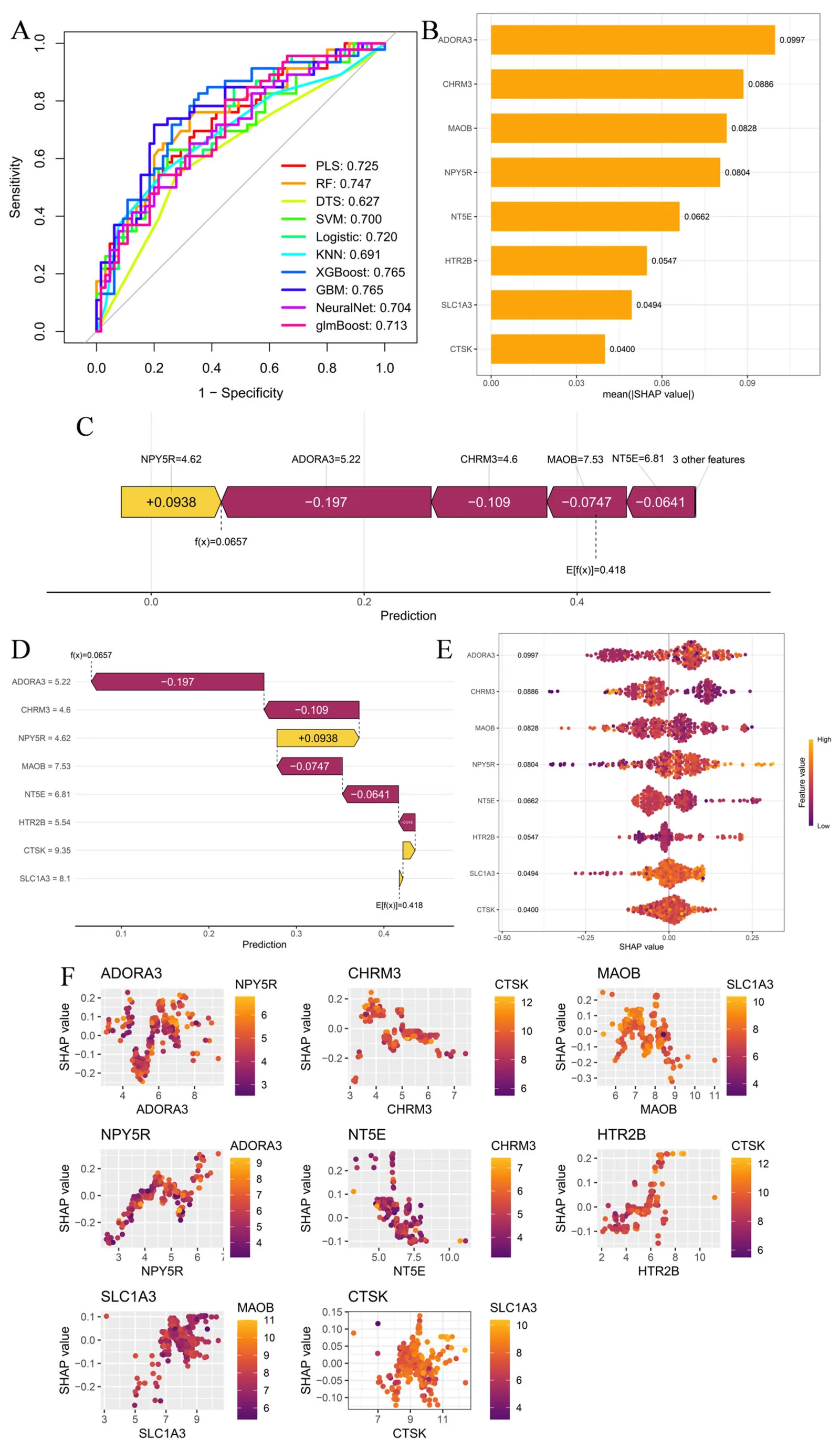
Interpretability of core genes using multi-model machine learning and SHAP analysis. (**A**) ROC curves of ten machine learning models for COPD classification. (**B**) Bar plot of mean absolute SHAP values for each gene, indicating their relative contributions to the model. (**C**) SHAP force plot showing feature contributions for an individual sample. (**D**) SHAP waterfall plot displaying the contribution of each gene to the prediction for a single sample. (**E**) SHAP summary (swarm) plot illustrating the distribution of SHAP values across samples; color represents gene expression level. (**F**) SHAP dependency plot showing the relationship between gene expression and corresponding SHAP values.

Ten machine-learning models were compared using the eight candidate genes. XGBoost and GBM produced the highest AUC in the integrated cohort (AUC = 0.765; Figure 3A), indicating moderate within-cohort discrimination. SHAP analysis was used to examine how individual genes contributed to model output. The mean absolute SHAP values ranked overall feature contributions (Figure 3B), whereas force and waterfall plots illustrated contributions for individual samples (Figure 3C,D). The SHAP swarm plot showed between-sample heterogeneity in feature effects (Figure 3E), and dependency plots suggested nonlinear relationships between gene expression and model output (Figure 3F). These findings support the interpretability of the fitted models; however, their predictive performance requires confirmation in an independent cohort.

### 2.4. Single-Gene GSEA and GSVA of Candidate-Gene-Associated Pathways

**Figure 4:**
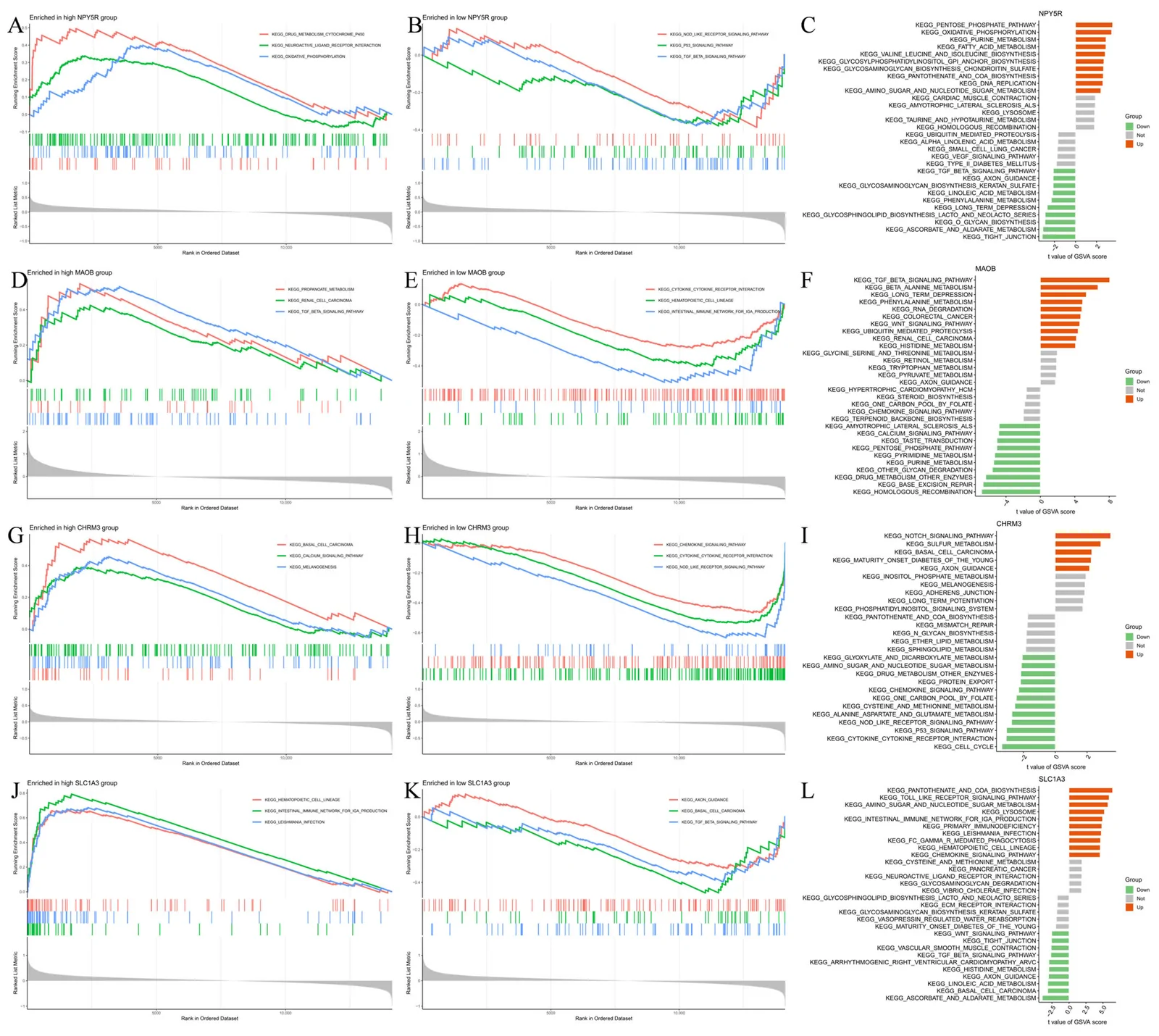
Functional enrichment analysis of core genes. (**A**,**B**,**D**,**E**,**G**,**H**,**J**,**K**) GSEA plots showing enrichment score distributions across ranked genes for high- and low-expression groups. (**C**,**F**,**I**,**L**) GSVA bar plots showing significantly enriched pathways in high- and low-expression groups; bar length represents statistics for comparisons of GSVA pathway scores, and color indicates the enrichment direction.

Single-gene GSEA and GSVA revealed distinct functional patterns among core genes (Figure 4A–L; additional results are shown in Supplementary Figure S2). GSEA showed that high expression of NT5E, CTSK, and MAOB was mainly enriched in glycolysis/gluconeogenesis, lysosome, extracellular matrix-receptor interaction, and focal adhesion pathways. In contrast, high expression of HTR2B, NPY5R, CHRM3, and SLC1A3 was primarily enriched in immune-related pathways, including cytokine-cytokine receptor interaction, neutrophil degranulation, T-cell receptor signaling, and TNF signaling. The low-expression group was enriched in pathways such as antigen processing and presentation, cell adhesion molecules, and neuroactive ligand–receptor interaction. GSVA results were consistent with GSEA findings, indicating that these core genes are associated with two major functional modules: metabolic/structural remodeling and inflammation/immune regulation.

### 2.5. Immune Infiltration and Gene–Immune Cell Correlations

**Figure 5:**
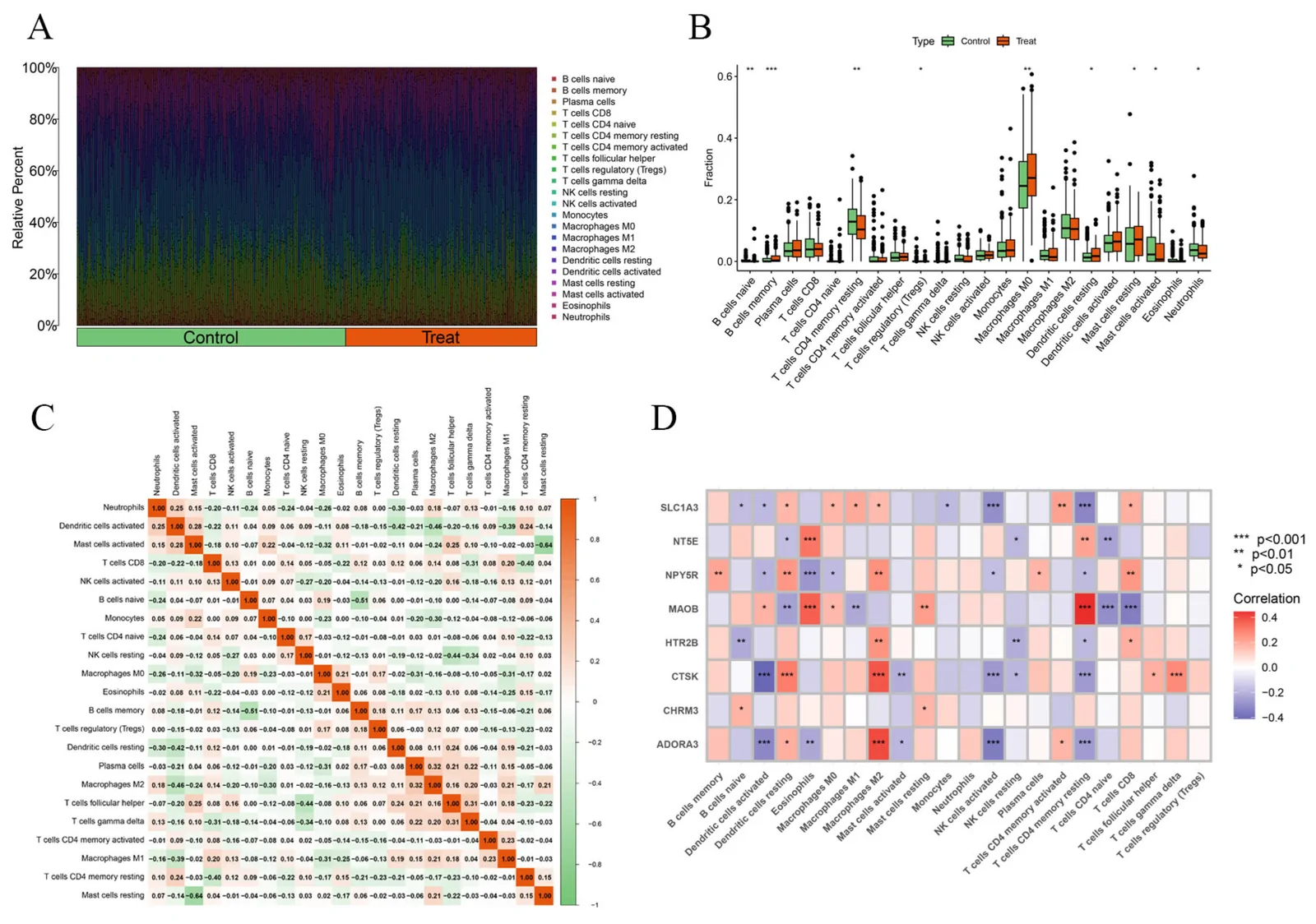
Immune infiltration characteristics and gene-immune cell correlations in COPD. (**A**) Stacked bar plot showing the relative proportions of immune cell subsets across samples. (**B**) Box plots comparing immune cell infiltration between COPD and control groups (* *p* < 0.05, ** *p* < 0.01, *** *p* < 0.001). (**C**) Spearman correlation heatmap among immune cell subsets; color indicates the correlation coefficient. (**D**) Correlation heatmap between core genes and immune cell infiltration; color represents the correlation coefficient.

Immune cell infiltration was estimated using the CIBERSORT algorithm, which quantified the relative abundances of 22 immune cell subsets (Figure 5A–D). Distinct differences in immune cell composition were observed between COPD and control samples, with considerable inter-sample variability (Figure 5A). A total of nine immune cell subsets differed significantly between groups, including naive B cells, memory B cells, resting CD4+ memory T cells, regulatory T cells, M0 macrophages, resting dendritic cells, resting mast cells, activated mast cells, and neutrophils (Figure 5B).

Correlation analysis revealed interactions among immune cell subsets, including a positive correlation between activated dendritic cells and neutrophils and a negative correlation between resting and activated mast cells (Figure 5C). Further analysis showed that several core genes were significantly associated with specific immune cell subsets. For example, SLC1A3 was positively correlated with M1 macrophages and activated CD4+ memory T cells, whereas NT5E was negatively correlated with naive CD4+ T cells (Figure 5D).

### 2.6. Single-Cell RNA-Sequencing Analysis

**Figure 6:**
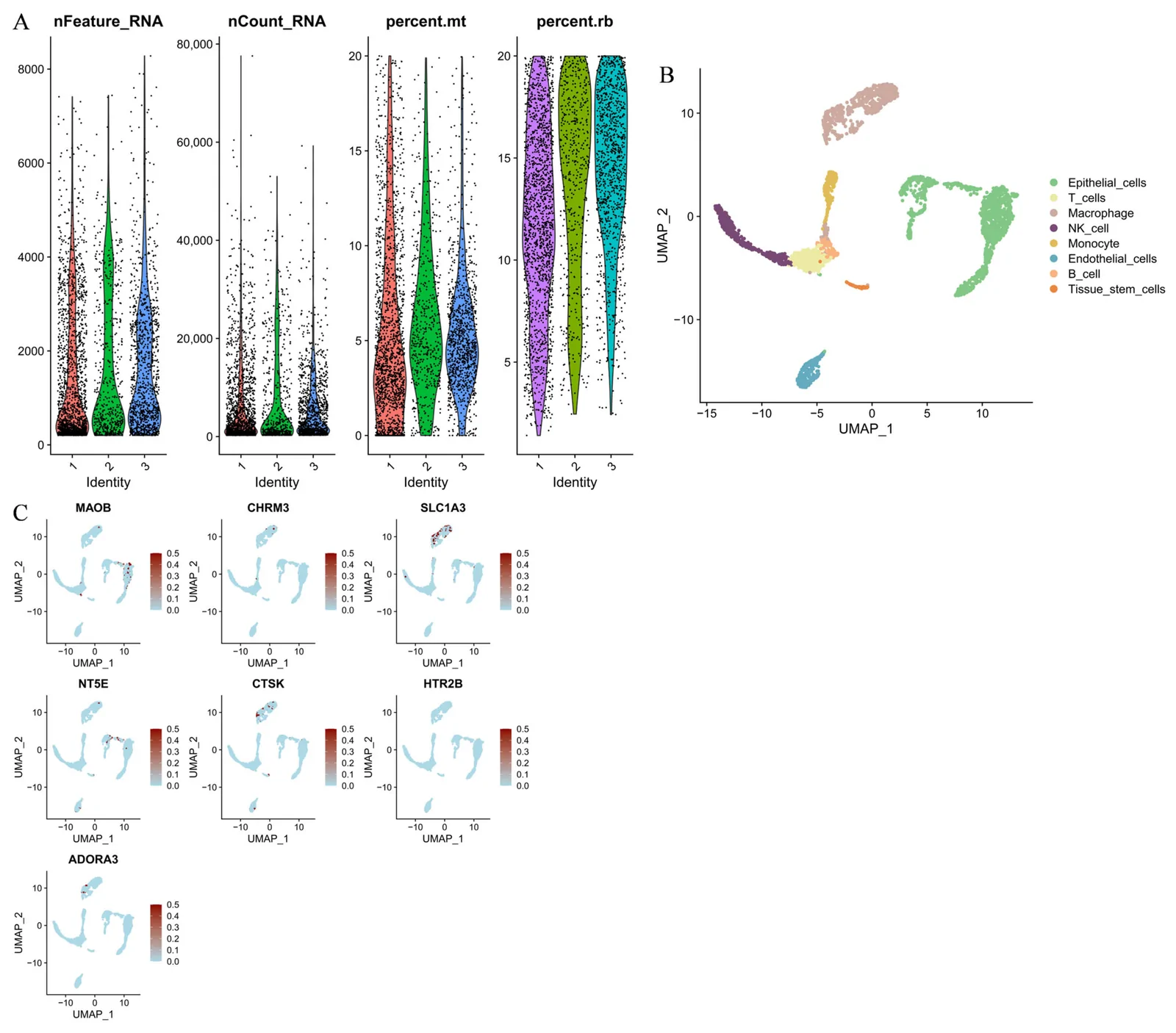
ScRNA-seq analysis and cell cluster identification. (**A**) Quality control metrics of scRNA-seq data, including nFeature_RNA, nCount_RNA, percent.mt, and percent.rb. (**B**) UMAP visualization showing transcriptionally distinct cell clusters; cell types were annotated based on canonical marker genes. (**C**) Feature plots showing the expression patterns of representative genes (MAOB, CHRM3, SLC1A3, NT5E, CTSK, HTR2B, and ADORA3) across cell clusters.

ScRNA-seq analysis was performed using the GSE167295 dataset (Figure 6A–C). Quality control indicated that most cells exhibited moderate gene and transcript counts with low mitochondrial gene content, supporting overall data reliability (Figure 6A). Dimensionality reduction and clustering identified multiple distinct cell clusters, indicating substantial cellular heterogeneity (Figure 6B). Based on SingleR annotation, these clusters were classified as epithelial cells, T cells, macrophages, NK cells, monocytes, endothelial cells, B cells, and tissue stem cells. Expression analysis showed that core genes, including MAOB, CHRM3, SLC1A3, NT5E, CTSK, HTR2B, and ADORA3, displayed cell-type-specific expression patterns across different clusters (Figure 6C).

### 2.7. Molecular Docking

**Figure 7:**
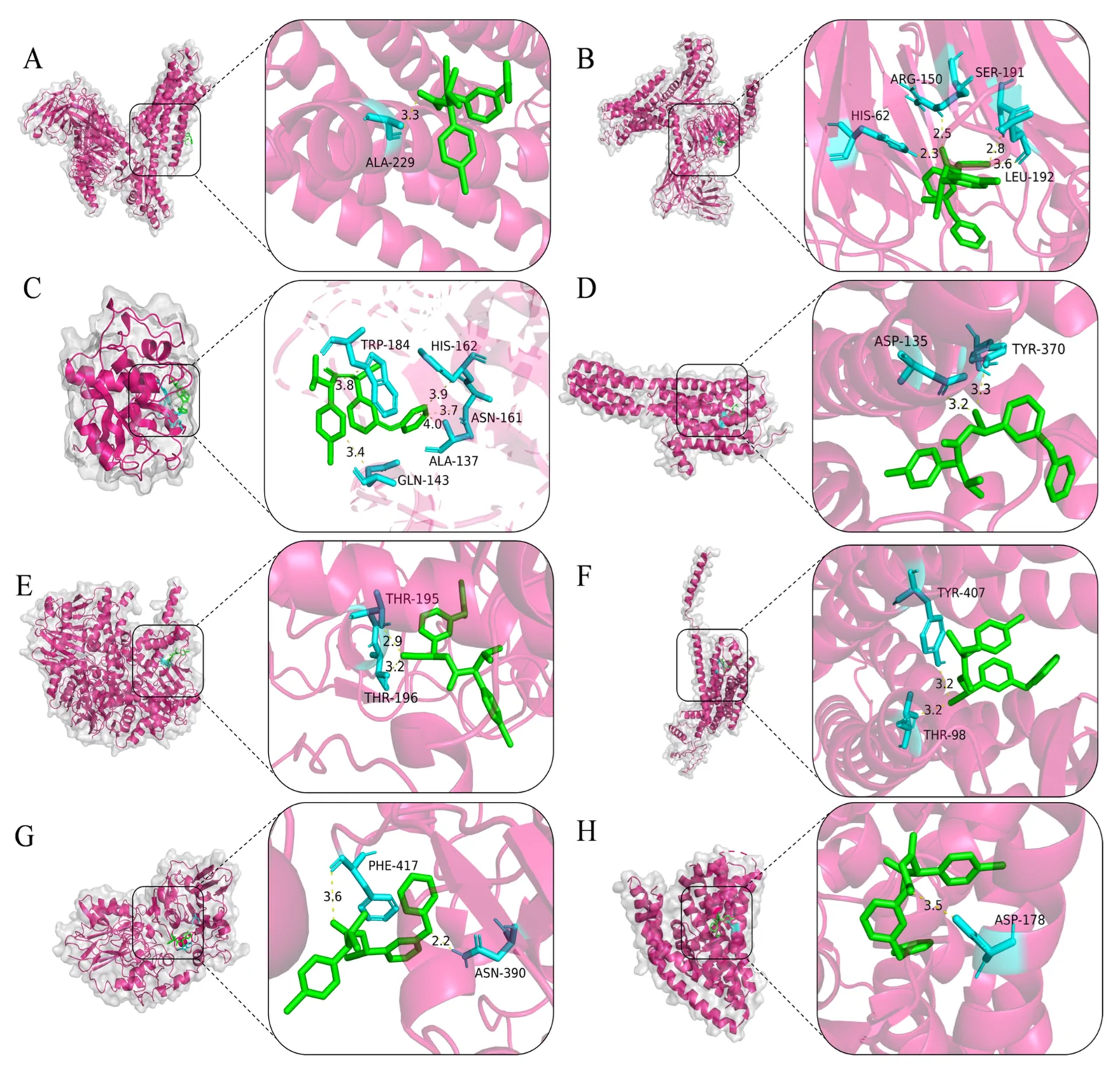
Visualization of molecular docking complexes between FEN and core targets. (**A**) FEN and ADORA3. (**B**) FEN and CHRM3. (**C**) FEN and CTSK. (**D**) FEN and HTR2B. (**E**) FEN and MAOB. (**F**) FEN and NPY5R. (**G**) FEN and NT5E. (**H**) FEN and SLC1A3.

Molecular docking was used to estimate potential interactions between FEN and the eight candidate proteins. All predicted binding energies were below −5.0 kcal/mol (Table 1), suggesting potentially favorable predicted ligand–protein interactions under the docking conditions employed. MAOB had the lowest predicted docking energy (−11.5 kcal/mol), followed by HTR2B, NT5E, SLC1A3, and NPY5R, indicating the most favorable predicted binding affinity among the eight candidate proteins under the docking conditions employed. The predicted poses placed FEN within putative binding pockets and suggested hydrogen-bond and hydrophobic contacts with surrounding residues (Figure 7). These docking scores and poses represent computational hypotheses and do not demonstrate direct physical binding.

### 2.8. Molecular Dynamics Simulation

**Figure 8:**
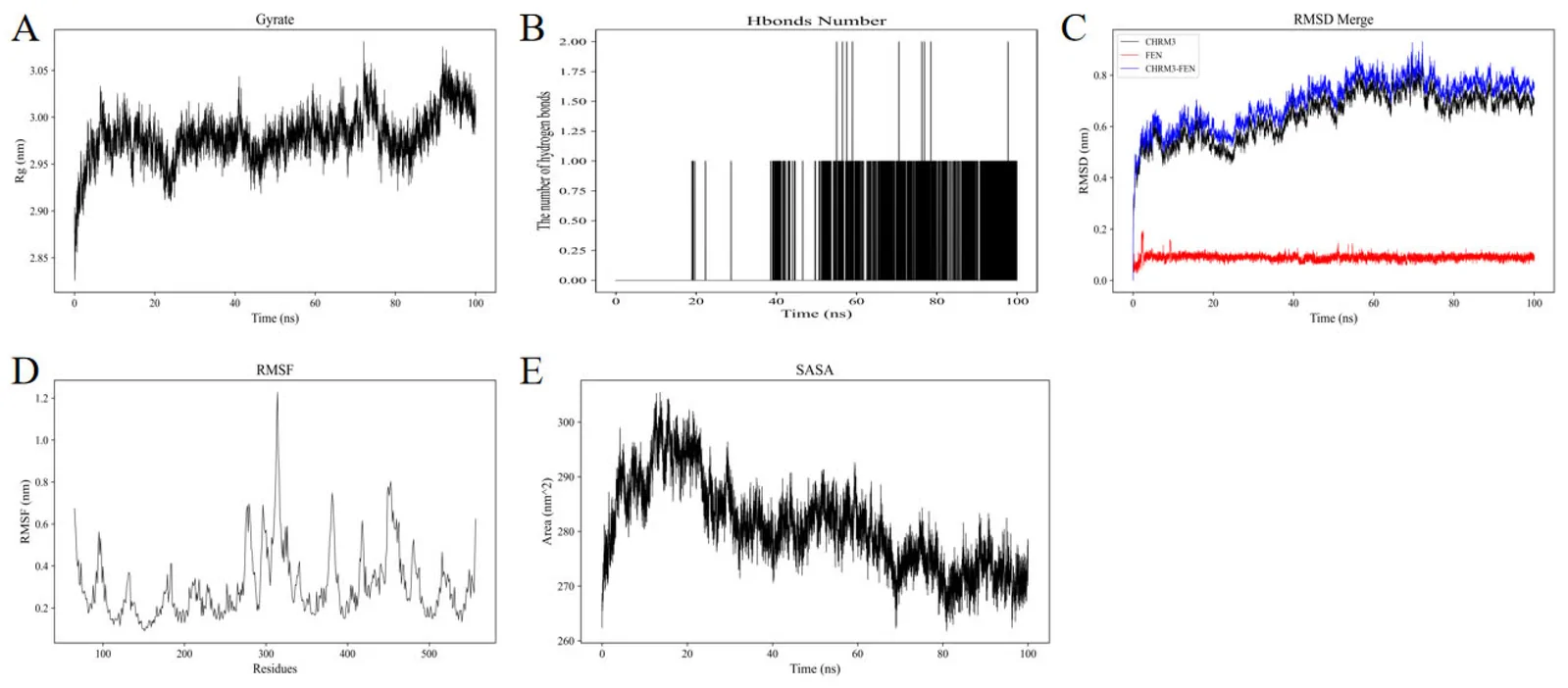
MD simulation analysis of the CHRM3-FEN complex. (**A**) Rg analysis. (**B**) Hydrogen bond analysis. (**C**) RMSD analysis. (**D**) RMSF analysis. (**E**) SASA analysis.

CHRM3 was selected for MD simulation because it was prioritized by all three feature-selection methods, associated with inflammation-related pathways, expressed in airway-related cell populations in the single-cell dataset, and predicted to interact with FEN (docking energy, −7.3 kcal/mol). During the 100 ns simulation, the radius of gyration remained approximately 2.95–3.05 nm (Figure 8A). The number of hydrogen bonds varied between zero and two over time (Figure 8B). Protein and complex RMSD values fluctuated mainly between 0.45 and 0.85 nm, while the ligand RMSD remained approximately 0.05–0.10 nm (Figure 8C). Residue-level fluctuations were generally limited, with greater flexibility around residues 280–310 (Figure 8D), and solvent-accessible surface area remained approximately 270–300 nm^2^ (Figure 8E). MM/GBSA calculations yielded a favorable estimated binding free energy dominated by van der Waals contributions (Table 2). Together, these simulations support the stability of the predicted pose under the modeled conditions but require biophysical confirmation.

### 2.9. Pharmacological Assessment of CHRM3/NF-κB Signaling in FEN-Induced Cellular Injury

In vitro experiments were used to examine whether CHRM3- and NF-κB-related signaling contributed to FEN-induced epithelial injury (Supplementary Figure S3). FEN reduced cell viability relative to the vehicle control (*p* < 0.001). CHRM3 inhibition alone did not significantly affect viability, whereas CHRM3 or NF-κB inhibition partially restored viability in FEN-treated cells. FEN also increased IL-6 and IL-8 secretion (*p* < 0.01), and both inhibitors attenuated these increases. Western blotting showed increased CHRM3 expression, higher p-IκBα/IκBα and p-p65/p65 ratios, reduced total IκBα, increased cleaved caspase-3, and reduced Bcl-2 after FEN exposure. CHRM3 inhibition attenuated NF-κB pathway activation and apoptosis-related protein changes. NF-κB inhibition reduced pathway activation and apoptosis markers without altering CHRM3 expression. Annexin V-FITC/PI staining confirmed increased apoptosis after FEN treatment (*p* < 0.001), which was partially reduced by either inhibitor. These pharmacological rescue experiments are consistent with CHRM3 acting upstream of NF-κB, but receptor-specific genetic perturbation is needed to establish pathway order and causality.

Collectively, FEN induced inflammatory and apoptotic responses in BEAS-2B cells, and pharmacological inhibition of CHRM3 or NF-κB partially attenuated these effects. The data therefore support involvement of CHRM3-associated NF-κB signaling in this cellular model without establishing that FEN causes COPD.

## 3. Discussion

COPD is a common chronic respiratory disorder characterized by persistent airflow limitation, chronic inflammation, and airway remodeling. Although cigarette smoking remains the primary risk factor, increasing evidence indicates that environmental chemical exposure also contributes to COPD pathogenesis [1]. FEN, a widely used PYR insecticide, exhibits environmental persistence and has been reported to induce pulmonary inflammation and structural injury; however, its association with COPD and the underlying molecular mechanisms remain unclear. Several previous toxicological studies have demonstrated that fenvalerate exposure can induce pulmonary inflammation, epithelial hypertrophy, alveolar edema, and biochemical evidence of lung injury in experimental animals. These studies, however, primarily characterized the pulmonary toxic effects of fenvalerate and did not address the molecular mechanisms that may link FEN exposure to COPD-related airway epithelial injury. In particular, FEN-associated molecular targets have not been systematically integrated with COPD-related transcriptomic alterations, cell-type-specific expression patterns, and experimental validation within a unified mechanistic framework. Therefore, the present study extends previous toxicological observations by investigating the molecular basis of FEN-associated airway epithelial injury in the context of COPD-related biological processes and by prioritizing a testable CHRM3-associated NF-κB signaling mechanism. Importantly, this study was designed to identify and experimentally evaluate a biologically plausible mechanism linking FEN exposure with airway epithelial injury rather than to establish that FEN directly causes COPD development.

This study combined network toxicology, bulk and single-cell transcriptomics, machine learning, molecular docking, MD simulation, and BEAS-2B experiments to examine a mechanistic link between FEN exposure and COPD-related airway epithelial injury. In this framework, computational analyses served as a hypothesis-generation and candidate-prioritization strategy, whereas cellular experiments were performed to provide functional support for the most biologically plausible mechanism identified from the integrated analyses. Through this integrated framework, CHRM3 emerged as a biologically plausible candidate that may mediate the association between FEN exposure and inflammatory and apoptotic responses in airway epithelial cells. The convergent computational and experimental findings collectively support CHRM3-associated NF-κB signaling as a plausible mechanistic pathway, while the observational and computational components remain hypothesis-generating.

At the bioinformatics level, the identified core genes were mainly enriched in pathways related to inflammatory responses, immune regulation, and neuroactive ligand-receptor interactions, suggesting that COPD progression involves not only classical inflammatory processes but also potential dysregulation of neural signaling and intercellular communication. Among these genes, CHRM3, a representative G protein-coupled receptor (GPCR), mediates acetylcholine signaling and plays a key role in airway smooth muscle contraction, mucus secretion, and airway hyperresponsiveness [18,19]. Previous studies have shown that CHRM3 activation can trigger Gq protein-mediated phospholipase C (PLC) signaling, leading to Ca^2+^ influx and protein kinase C (PKC) activation, thereby promoting downstream inflammatory pathways, including NF-κB [20,21]. In addition, CHRM3 has been implicated in inflammatory cell recruitment and cytokine release, contributing to chronic airway inflammation [22,23]. Importantly, CHRM3 is highly expressed in airway smooth muscle and epithelial cells, where excessive cholinergic signaling has been implicated in airway hyperresponsiveness, mucus hypersecretion, and inflammatory amplification, all of which are characteristic features of COPD.

CHRM3 was prioritized across the feature-selection analyses, was associated with inflammation-related pathways, showed cell-type-specific expression in the single-cell dataset, and had a favorable predicted docking energy with FEN. On this basis, CHRM3 was selected for MD simulation. Docking and MD analyses supported a potentially stable interaction under the modeled conditions, but neither method establishes direct binding. In BEAS-2B cells, FEN reduced viability, increased IL-6 and IL-8 secretion, and promoted apoptosis, demonstrating cytotoxic and pro-inflammatory effects in the experimental model.

FEN exposure was associated with increased CHRM3 abundance and activation of NF-κB signaling, reflected by increased IκBα and p65 phosphorylation and reduced total IκBα. CHRM3 inhibition partially attenuated NF-κB activation and cellular injury, whereas direct NF-κB inhibition exerted a stronger downstream effect [24]. These pharmacological findings are consistent with, but do not prove, a model in which CHRM3 contributes to upstream regulation of NF-κB signaling after FEN exposure. Receptor knockdown or knockout, together with direct target-engagement assays, will be required to establish specificity and pathway order.

Notably, neither CHRM3 nor NF-κB inhibition fully restored cellular function, suggesting that FEN-induced cytotoxicity may involve multiple signaling pathways. This is consistent with the multifactorial and complex pathogenesis of COPD [25]. Furthermore, immune infiltration and single-cell analyses revealed close associations between core genes and multiple immune cell subsets, indicating that FEN may exacerbate inflammation by modulating the immune microenvironment. Given the importance of immune dysregulation in COPD, these findings provide additional support for the interplay between inflammation and immune imbalance in disease progression [26].

Several limitations should be considered. Importantly, the current findings represent an integrated mechanistic investigation supported by targeted cellular validation, rather than a complete demonstration of disease causality. First, the experimental validation used a single immortalized bronchial epithelial cell line and did not include primary airway cells, air–liquid interface cultures, animal models, or direct exposure assessment in humans. Second, the integrated bulk-transcriptomic analysis combined datasets generated on different platforms, and candidate selection and model performance were not confirmed in an independent external cohort. Third, predicted targets, docking, and MD simulations are hypothesis-generating and do not demonstrate direct FEN–CHRM3 engagement. Fourth, scopolamine is not selective for CHRM3, and pharmacological inhibition alone cannot establish receptor specificity or pathway order. Finally, the 0.5 μM repeated-exposure regimen provides a cellular hazard model but should not be interpreted as a direct estimate of environmentally relevant airway dose. Future studies should include genetic perturbation of CHRM3, biophysical target-engagement assays, primary airway models, and in vivo exposure–response validation.

In summary, FEN elicited inflammatory and apoptotic responses in bronchial epithelial cells, and these effects were partially attenuated by pharmacological inhibition of CHRM3 or NF-κB. Integrated transcriptomic and computational analyses further identified CHRM3 as a plausible mechanistic candidate. The findings support a testable model of CHRM3-associated NF-κB activation in FEN-induced airway epithelial injury; however, confirmation in primary airway models, animal studies, and direct target-engagement assays will be required before drawing conclusions regarding physiological relevance, therapeutic implications, or COPD causation.

## 4. Materials and Methods

### 4.1. Network Toxicology Analysis

#### 4.1.1. Identification and Enrichment Analysis of Potential FEN Targets and COPD-Associated Differentially Expressed Genes

The canonical SMILES string for FEN was retrieved from PubChem [27] (https://pubchem.ncbi.nlm.nih.gov/, accessed on 29 November 2025) and submitted to SwissTargetPrediction [28] (http://www.swisstargetprediction.ch/, accessed on 29 November 2025), the Similarity Ensemble Approach database [29] (https://sea.bkslab.org/, accessed on 29 November 2025), and SuperPred [30] (http://prediction.charite.de, accessed on 29 November 2025), with the species restricted to Homo sapiens. For SwissTargetPrediction, predicted targets with a probability greater than 0 were retained. For the Similarity Ensemble Approach database and SuperPred, all available Homo sapiens protein targets returned by the respective platforms were included without additional filtering criteria. Predicted targets were pooled, duplicates were removed, and gene/protein identifiers were standardized using UniProt [31] (https://www.uniprot.org/, accessed on 29 November 2025).

COPD-related gene expression datasets, including GSE130928, GSE20257, and GSE76925, were retrieved from the GEO database [32] (https://www.ncbi.nlm.nih.gov/geo/, accessed on 30 November 2025). These three independent human COPD transcriptomic datasets were selected based on the following criteria: (i) inclusion of clinically diagnosed COPD patients together with appropriate control groups; (ii) availability of pulmonary compartment-derived transcriptomic data relevant to COPD pathobiology; (iii) complete gene expression matrices with corresponding phenotype information; and (iv) sufficient sample sizes for comparative and integrated analyses. Specifically, GSE130928 represents alveolar macrophages isolated from bronchoalveolar lavage fluid, GSE20257 profiles small airway epithelium, and GSE76925 profiles lung tissue, thereby capturing complementary pulmonary compartments involved in COPD pathogenesis. Integrating these datasets was intended to identify COPD-associated molecular signatures that are consistently observed across biologically relevant pulmonary compartments, thereby reducing dependence on findings derived from a single dataset. To improve the robustness and comparability of the integrated analysis, normalization and batch-effect correction were performed before downstream analyses. Batch effects were corrected using the ComBat algorithm implemented in the sva package in R (version 4.3.3) [33] to account for platform heterogeneity. Principal component analysis (PCA) was performed before and after batch-effect correction to evaluate the effectiveness of data integration. After duplicate samples and low-quality probe annotations were removed, the datasets were merged into a single cohort. Differential expression analysis was performed using the limma package in R [34]. To control for multiple testing, P values were adjusted using the Benjamini–Hochberg false discovery rate (FDR) method. Genes with |log2 fold change (FC)| > 0.3 and an adjusted *p* value < 0.05 were considered COPD-associated differentially expressed genes (DEGs).

The overlap between predicted FEN targets and COPD-associated DEGs was identified using Venny (version 2.1). Gene Ontology (GO) and Kyoto Encyclopedia of Genes and Genomes (KEGG) enrichment analyses were performed with clusterProfiler (version 4.10.1) [35]. Enrichment results with adjusted *p* < 0.05 were considered statistically significant.

#### 4.1.2. Machine Learning for Core Target Screening

Three machine-learning methods were used for feature selection: least absolute shrinkage and selection operator (LASSO) regression, random forest (RF), and support vector machine (SVM) [36]. LASSO was implemented with glmnet (version 4.1-8), and ten-fold cross-validation was used to select the penalty parameter (λ) [37]. The RF model was fitted with randomForest using 500 trees, and feature importance was assessed using mean decrease in Gini impurity [38]. The SVM was implemented with e1071 using a radial basis function kernel, with C and γ tuned during model fitting [39]. Genes retained by all three methods were designated candidate genes. Because selection and performance assessment were conducted in the integrated cohort, this procedure was exploratory and did not constitute external validation.

#### 4.1.3. Expression, Chromosomal Localization, and Correlation Analysis of Core Genes

The expression levels of core genes were extracted from normal and COPD samples, and differential expression analysis was performed using the limma package in R [34]. Core genes with *p* < 0.05 were considered significantly differentially expressed, and their expression patterns were visualized using box plots and heatmaps. Chromosomal localization was determined using a Perl script and visualized using the RCircos (version 1.2.2) package [40]. Pearson correlation analysis was performed among differentially expressed core genes, and correlation patterns were displayed as heatmaps.

#### 4.1.4. Multi-Model Comparison and SHAP Interpretability Analysis

To evaluate the predictive value of differentially expressed core genes, ten machine learning models were constructed, including partial least squares (PLS), RF, decision tree (DT), SVM, logistic regression, k-nearest neighbors (KNN), extreme gradient boosting (XGBoost), gradient boosting machine (GBM), neural network, and generalized linear model boosting (glmBoost). These models provide complementary strengths for modeling linear and nonlinear relationships in high-dimensional data. XGBoost, an optimized gradient boosting method, enables efficient parallel computation and robust handling of missing data [41]. Model performance was evaluated based on the area under the receiver operating characteristic (ROC) curve (AUC), sensitivity, specificity, and accuracy. The model with the best overall predictive performance was selected for subsequent analyses. SHapley Additive exPlanations (SHAP) analysis was then applied to quantify feature contributions and determine their effects on classification outcome [42].

#### 4.1.5. Functional Enrichment and Pathway Activity Analysis

Gene Set Enrichment Analysis (GSEA) and Gene Set Variation Analysis (GSVA) were performed to explore pathways associated with the identified core genes. For each core gene, samples were divided into high- and low-expression groups according to the median expression level. Genes were ranked according to their association with the corresponding core gene, and GSEA was conducted using the clusterProfiler package with curated pathway gene sets from MSigDB as the reference. Pathways with |NES| > 1 and *p* < 0.05 were considered significantly enriched. GSVA was performed using the GSVA package to calculate sample-level pathway activity scores, which were subsequently compared between the high- and low-expression groups to identify pathway activity patterns associated with core-gene expression.

#### 4.1.6. Immune Infiltration Analysis and Correlation Analysis

CIBERSORT (version 1.03) was used to estimate the relative proportions of immune cell types in the integrated GEO dataset, with 1000 permutations used to ensure statistical robustness [43]. Stacked bar plots were generated to show immune cell composition, and single-sample Gene Set Enrichment Analysis (ssGSEA) was performed to evaluate immune infiltration levels [44]. Differences between normal and COPD groups were assessed using the Wilcoxon rank-sum test. Spearman correlation analysis was used to evaluate associations between core gene expression and immune-related features, and results were visualized as heatmaps.

#### 4.1.7. ScRNA-Seq Analysis

ScRNA-seq data from the COPD-related dataset GSE167295 were retrieved from GEO and analyzed in R. The Seurat package was used for quality control, normalization, dimensionality reduction, and clustering [45]. Batch effects were corrected using Harmony [46], and Uniform Manifold Approximation and Projection (UMAP) was used for visualization [47]. Cell types were annotated using SingleR [48]. Gene set activity across cell populations was evaluated using UCell [49], and rank-based single-cell gene set enrichment analysis was performed using irGSEA [50].

#### 4.1.8. Molecular Docking Analysis

For protein–ligand interaction analysis, three-dimensional protein structures were obtained from the Protein Data Bank (PDB) [51] (https://www.rcsb.org/, accessed on 23 January 2026) and the FEN structure was obtained from PubChem. Protein structures were prepared by removing crystallographic water molecules and co-crystallized ligands, adding polar hydrogens, and assigning charges. Docking was performed with AutoDock Vina (version 1.2.3), and predicted poses were ranked by docking energy. In AutoDock Vina, the predicted binding energy represents the estimated free energy of ligand–receptor binding, with more negative values indicating stronger predicted binding affinity. Accordingly, docking scores below −5.0 kcal/mol were considered indicative of potentially favorable predicted interactions, whereas lower (more negative) scores suggest relatively stronger predicted binding affinities [52]. Binding conformations were visualized using PyMOL (version 2.6) to analyze key residues and noncovalent interactions.

#### 4.1.9. MD Simulations

To further validate protein-ligand binding stability, molecular dynamics (MD) simulations were performed for the CHRM3-FEN complex based on docking, enrichment, and scRNA-seq results [53,54]. FEN was parameterized using AmberTools v22 with the General Amber Force Field (GAFF). Geometry optimization and electrostatic potential calculations were performed using Gaussian 16W, and restrained electrostatic potential (RESP) charges were incorporated into the system topology. Simulations were conducted under periodic boundary conditions at 300 K and 1 bar. The Amber99SB-ILDN force field was applied to the protein, and the system was solvated using the TIP3P water model. Na+ ions were added to neutralize the total system charge. Energy minimization was performed using the steepest descent algorithm, followed by NVT and NPT equilibration for 100 ps each. A 100 ns production simulation was then conducted with a 2 fs time step. Trajectories were analyzed for root-mean-square deviation (RMSD), root-mean-square fluctuation (RMSF), radius of gyration (Rg), and Molecular Mechanics Generalized Born Surface Area (MM/GBSA) binding free energy.

### 4.2. Cell Experiments

#### 4.2.1. Reagents

BEAS-2B human bronchial epithelial cells were obtained from the American Type Culture Collection (ATCC, Manassas, VA, USA). FEN and dimethyl sulfoxide were purchased from Sigma-Aldrich (St. Louis, MO, USA). The CHRM3 inhibitor scopolamine and the NF-κB inhibitor BAY 11-7082 were obtained from MedChemExpress (Monmouth Junction, NJ, USA). RPMI 1640 medium, fetal bovine serum, and penicillin-streptomycin solution were purchased from Gibco (Grand Island, NY, USA). The Cell Counting Kit-8 was obtained from Dojindo (Mashiki, Kumamoto, Japan). The Annexin V-FITC/PI apoptosis detection kit, 1× binding buffer, and ELISA kits for interleukin-6 and interleukin-8 were purchased from Elabscience (Wuhan, China). Cells were cultured in 96-well or 6-well plates from Corning (Corning, NY, USA).

#### 4.2.2. Cell Culture and Treatment

BEAS-2B cells were cultured in complete medium supplemented with fetal bovine serum and penicillin-streptomycin and maintained at 37 °C in a humidified incubator containing 5% CO_2_. The FEN concentration was selected based on previous in vitro toxicological studies and preliminary CCK-8 experiments. Cells were exposed to FEN at 0.5 μM for subsequent experiments, which induced measurable cellular injury without excessive cytotoxicity. The experimental groups included the control group, FEN-treated group, CHRM3 inhibitor group, FEN + CHRM3 inhibitor group, and FEN + NF-κB inhibitor group. FEN exposure was performed using a repeated low-dose regimen. Briefly, the medium was replaced with fresh FEN-containing medium every 24 h for three consecutive days to better mimic environmental exposure. For inhibitor experiments, cells were pretreated with the CHRM3 inhibitor or NF-κB inhibitor for 1 h before each FEN exposure, and inhibitors were maintained in the culture medium during subsequent treatment. The final dimethyl sulfoxide concentration was kept consistent across groups and maintained below 0.1%.

#### 4.2.3. CCK-8 Assay

Cell viability was assessed using the CCK-8 assay as previously described [55]. Briefly, BEAS-2B cells were seeded in 96-well plates at a density of 5 × 10^3^ cells/well and cultured for 24 h. The cells were subsequently exposed to FEN at a concentration of 0.5 μM. Fresh medium containing 0.5 μM FEN was replaced every 24 h for three consecutive days. Control cells received an equivalent volume of vehicle. After the final exposure, 10 μL of CCK-8 reagent was added to each well, and the plates were incubated at 37 °C for 2 h in the dark. Absorbance was measured at 450 nm using a microplate reader (Thermo Fisher Scientific, Waltham, MA, USA), and cell viability was expressed as a percentage of the control group.

#### 4.2.4. ELISA

IL-6 and IL-8 levels in cell culture supernatants were measured using ELISA kits according to the manufacturer’s instructions, as previously described [56]. Following the indicated treatments, cell culture supernatants were collected and centrifuged to remove cell debris before analysis. Standards and samples were then added to antibody-precoated microplates, and the assay was performed according to the manufacturer’s protocol. Cytokine concentrations were determined from standard curves generated with the supplied standards. Absorbance was measured at 450 nm using a microplate reader, and IL-6 and IL-8 concentrations were calculated based on the corresponding standard curves. All samples were assayed in triplicate.

#### 4.2.5. Western Blotting

Protein expression was analyzed by Western blotting as previously described [57]. Briefly, total proteins were extracted, separated by SDS-PAGE, and transferred onto membranes. Membranes were incubated with primary antibodies against CHRM3, p-IκBα, IκBα, p-p65, p65, cleaved caspase-3, and Bcl-2, followed by appropriate secondary antibodies. Signals were visualized using enhanced chemiluminescence and quantified using ImageJ (version 1.54g).

#### 4.2.6. Flow Cytometry Analysis

Apoptosis was assessed using Annexin V-FITC/PI staining according to the manufacturer’s instructions and previous studies [58]. Cells were collected, washed with phosphate-buffered saline, resuspended in binding buffer, and stained with Annexin V-FITC and PI for 15 min in the dark. Samples were analyzed by flow cytometry, and data were processed using FlowJo (version 10.8.1).

#### 4.2.7. Statistical Analysis

All experiments were performed in triplicate. Data are presented as the mean ± standard deviation. Statistical analyses were performed using SPSS 26.0. Multiple-group comparisons were conducted using one-way analysis of variance followed by Tukey’s post hoc test. Dunnett’s T3 test was used when variances were unequal. A *p* value < 0.05 was considered statistically significant.

## 5. Conclusions

By integrating predicted FEN targets, bulk and single-cell transcriptomics, machine learning, molecular modeling, and BEAS-2B experiments, this study identified CHRM3 as a plausible contributor to FEN-associated airway epithelial injury. Docking and MD simulations supported a potentially stable predicted FEN–CHRM3 interaction, while repeated 0.5 μM FEN exposure reduced cell viability, increased inflammatory cytokine secretion, and promoted apoptosis. CHRM3 or NF-κB inhibition partially attenuated these responses. The results support involvement of CHRM3-associated NF-κB signaling in this cellular model but do not establish direct FEN–CHRM3 binding or a causal role for FEN in COPD. Genetic, biophysical, primary-cell, and in vivo validation is warranted.

## Figures and Tables

**Table 1:** Predicted molecular docking energies for FEN and the eight candidate proteins.

Target	Binding Energy (kcal/mol)
ADORA3	−6.4
CHRM3	−7.3
CTSK	−6.6
HTR2B	−9.8
MAOB	−11.5
NPY5R	−7.7
NT5E	−8.9
SLC1A3	−8.1

**Table 2:** MM/GBSA energy components for the predicted CHRM3–FEN complex (kcal/mol; mean ± SD).

Contribution Components	CHRM3-FEN
ΔVDWAALS	−51.53 ± 0.49
ΔEelec	−3.33 ± 1.22
ΔEGB	24.36 ± 0.57
ΔEsurf	−6.76 ± 0.11
ΔGgas	−54.86 ± 1.32
ΔGsolvation	17.59 ± 0.58
ΔTotal	−37.27 ± 1.44

## Data Availability

The publicly available transcriptomic datasets analyzed in this study are available from the NCBI Gene Expression Omnibus (https://www.ncbi.nlm.nih.gov/geo/, accessed on 30 November 2025) under accession numbers GSE130928, GSE20257, GSE76925, and GSE167295. The original contributions generated in this study are included in the article and Supplementary Materials. Additional processed data, analysis outputs, molecular-modeling files, and uncropped experimental images are available from the corresponding authors upon reasonable request.

## Supplementary Materials

The following supporting information can be downloaded at https://www.mdpi.com/article/10.3390/ijms27209201/s1.

## Abbreviations

The following abbreviations are used in this manuscript:

COPD	Chronic obstructive pulmonary disease
PYRs	Pyrethroid insecticides
FEN	Fenvalerate
GEO	Gene Expression Omnibus
ScRNA-seq	Single-cell RNA sequencing
FC	Fold change
DEGs	Differentially expressed genes
GO	Gene Ontology
KEGG	Kyoto Encyclopedia of Genes and Genomes
LASSO	Least Absolute Shrinkage and Selection Operator
RF	Random Forest
SVM	Support Vector Machine
DT	Decision tree
KNN	K-nearest neighbors
XGBoost	Extreme gradient boosting
GBM	Gradient boosting machine
GlmBoost	Generalized linear model boosting
ROC	Receiver operating characteristic
AUC	Area under the ROC curve
SHAP	SHapley Additive exPlanations
GSEA	Gene Set Enrichment Analysis
GSVA	Gene Set Variation Analysis
SsGSEA	Single-sample Gene Set Enrichment Analysis
UMAP	Uniform Manifold Approximation and Projection
PDB	Protein Data Bank
MD	Molecular dynamics
GAFF	General Amber Force Field
RESP	Restrained electrostatic potential
RMSD	Root-mean-square deviation
RMSF	Root-mean-square fluctuation
Rg	Radius of gyration
MM/GBSA	Molecular Mechanics Generalized Born Surface Area
PCA	Principal component analysis
PLC	Phospholipase C
PKC	Protein kinase C
