# Supplementary material for: Fenvalerate Induces Airway Epithelial Injury Through CHRM3-Associated NF-κB Activation
Source: Int J Mol Sci. 2026 Oct 16;27(20):9201. doi: 10.3390/ijms27209201 (PMC13705351; doi:10.3390/ijms27209201)
Supplement: Supplementary file 1 [file ijms-27-09201-s001.zip › Supplementary Figures.pdf]

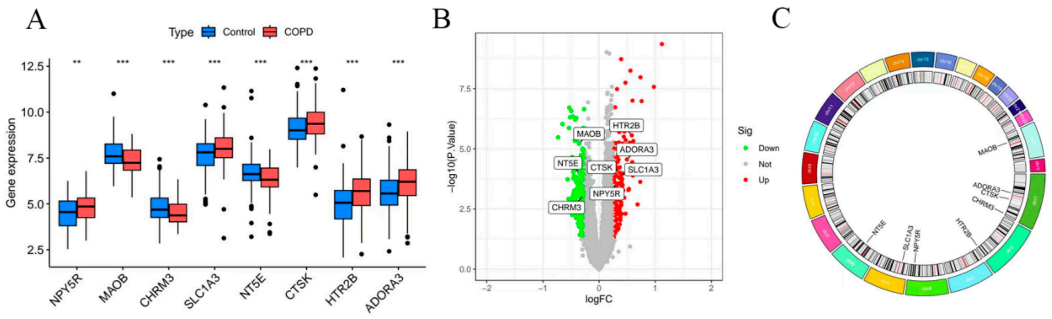

Supplementary Figure S1. Differential expression analysis and chromosomal localization of core genes. **(A)** Box plots showing the expression levels of intersecting genes in control and experimental groups. **(B)** Volcano plot showing differentially expressed genes. **(C)** Chromosomal distribution of intersecting genes.

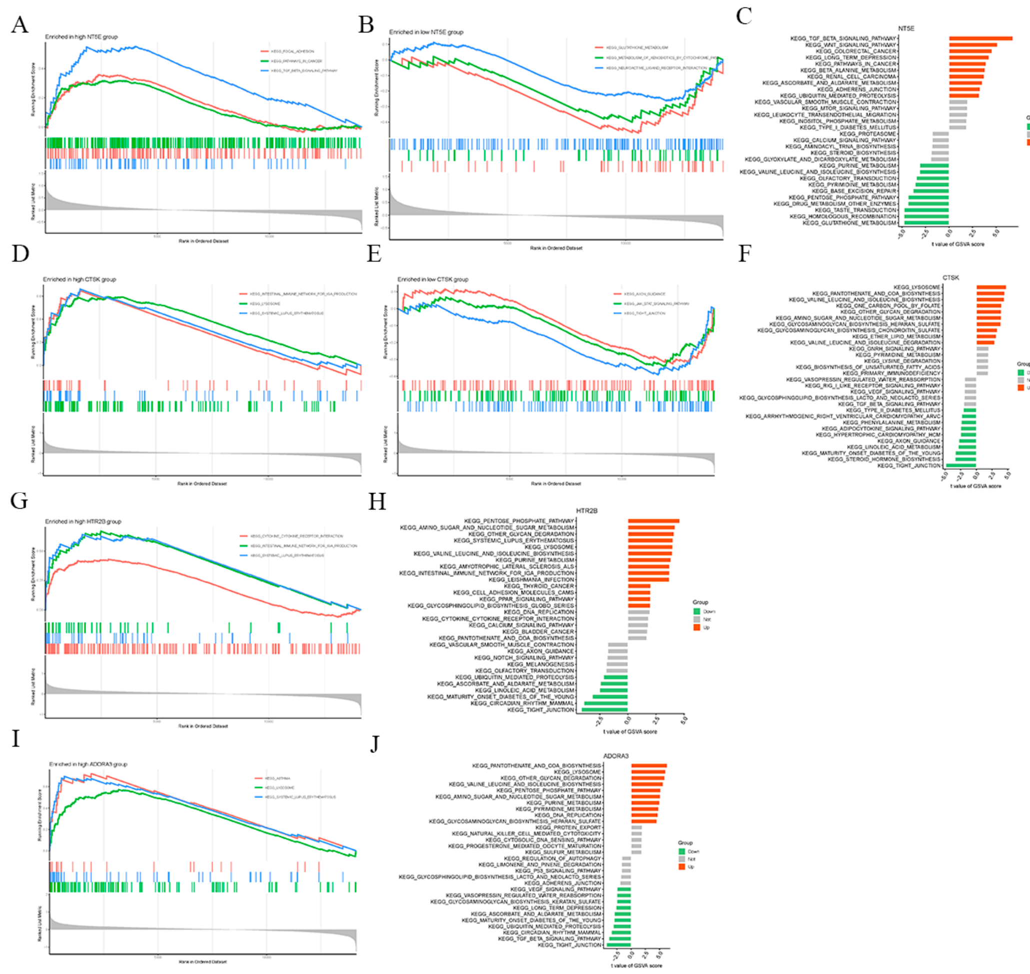

Supplementary Figure S2. Functional enrichment analysis of core genes. **(A-B, D-E, G, I)** GSEA plots showing enrichment score distributions across ranked genes for the high- and low-expression groups of NT5E, CTSK, HTR2B, and ADORA3, respectively. **(C, F, H, J)** GSVA bar plots showing significantly enriched pathways in the high- and low-expression groups; bar length represents the enrichment score, and color indicates the enrichment direction.

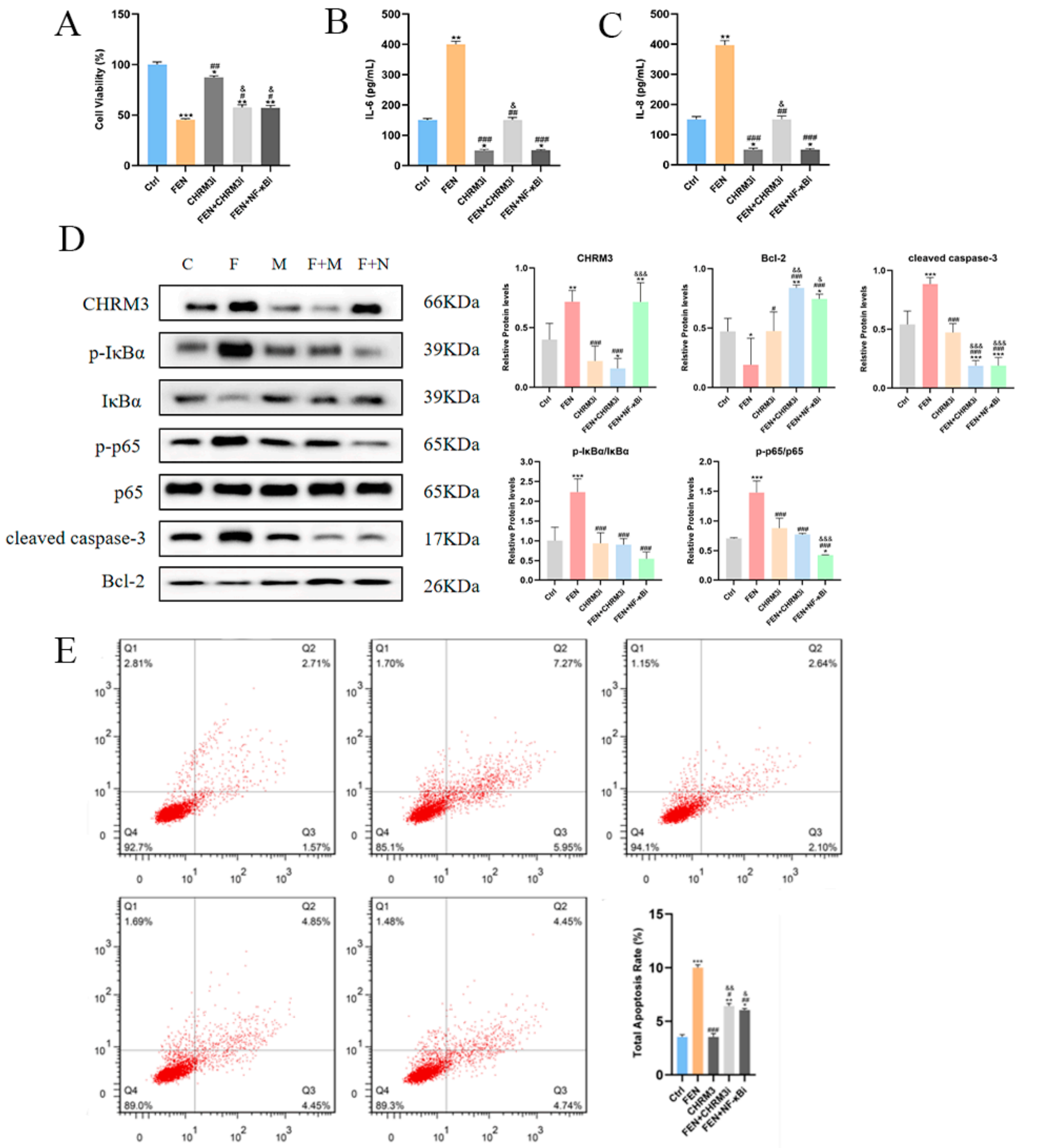

Supplementary Figure S3. Experimental validation of CHRM3/NF-κB signaling pathway involvement in FEN-induced cellular injury. **(A)** Cell viability analysis showing the effects of FEN, CHRM3 inhibition, and NF-κB inhibition on cell survival. **(B-C)** Effects of CHRM3 and NF-κB inhibition on FEN-induced secretion of inflammatory cytokines, including IL-6 and IL-8. **(D)** Western blotting analysis of CHRM3, NF-κB signaling-related proteins (p-IκBα, IκBα, p-p65, and p65), and apoptosis-associated proteins (cleaved caspase-3 and Bcl-2). Quantitative analyses of protein expression levels and phosphorylation ratios (p-IκBα/IκBα and p-p65/p65) are presented. **(E)** Flow cytometry analysis of apoptosis using Annexin V-FITC/PI staining and quantitative analysis of total apoptosis rates among different treatment groups. Data are presented as mean ± SD. *$p < 0.05$, **$p < 0.01$, ***$p < 0.001$ vs. Ctrl group; #$p < 0.05$, ##$p < 0.01$, ###$p < 0.001$ vs. FEN group; &$p < 0.05$, &&$p < 0.01$, &&&$p < 0.001$ vs. CHRM3 inhibitor group.
